# Quantitative imaging strategies pave the way for testable biological concepts

**DOI:** 10.1186/1741-7007-9-10

**Published:** 2011-02-25

**Authors:** Olivier Hamant

**Affiliations:** 1 Laboratoire Reproduction et Développement des Plantes/Laboratoire Joliot Curie, INRA, CNRS, ENS, Université de Lyon, 46 Allée d'Italie, 69364 Lyon Cedex 07, France

## Abstract

In developmental biology, the accumulation of qualitative phenotypic descriptions has fueled the need for testable parsimonious hypotheses, giving a fresh impetus to quantitative strategies. As an illustration, thanks to the precise quantification of cell growth and microtubule behavior in a study published in *BMC Plant Biology*, Zhang and collaborators have identified sequential phases of polarized and isotropic growth in puzzle-shaped leaf epidermal cells, thus providing new clues to explore how growth coordination occurs in this tissue.

## Quantification and hypothesis-driven science

**Figure 1:**
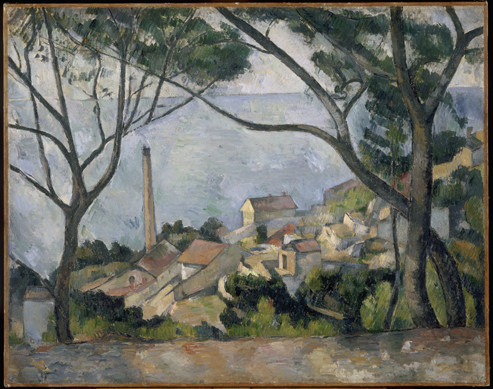
**Beyond qualitative imaging**. In Paul Cézanne's 'La mer à l'Estaque', trees can easily be recognized in the foreground. Nevertheless, a closer look reveals that the dimensions and geometry of the branches are not realistic at all, illustrating the relative weakness of the human eye and how a qualitative observation can lead to misleading conclusions. Reproduced with permission from Réunion des Musées Nationaux, Paris, France.

The qualitative description of developmental mutants has produced many famous theoretical biological concepts, but this approach reaches its limits when formally trying to establish links of causality between molecular networks and multicellular shape. In particular, there is an obvious discrepancy between the single-cell genetic input and the multicellular geometrical output. To bridge this gap, and prove that an intuition is plausible, a mathematical proof, usually in the form of computer simulations, becomes necessary. In this framework, an observation must be quantitative enough to be transposed into an algorithm. Conversely, to know whether a mathematical model is plausible, it must be validated experimentally, and this validation requires quantification of the observations [1] (Figure 1). Quantitative approaches have thus flourished in recent years, and they not only refine previous predictions but also generate novel hypotheses. The recent study by Zhang *et al. *in *BMC Plant Biology *[2] illustrates this nicely.

## The regulation and geometry of growth in leaf epidermis

**Figure 2:**
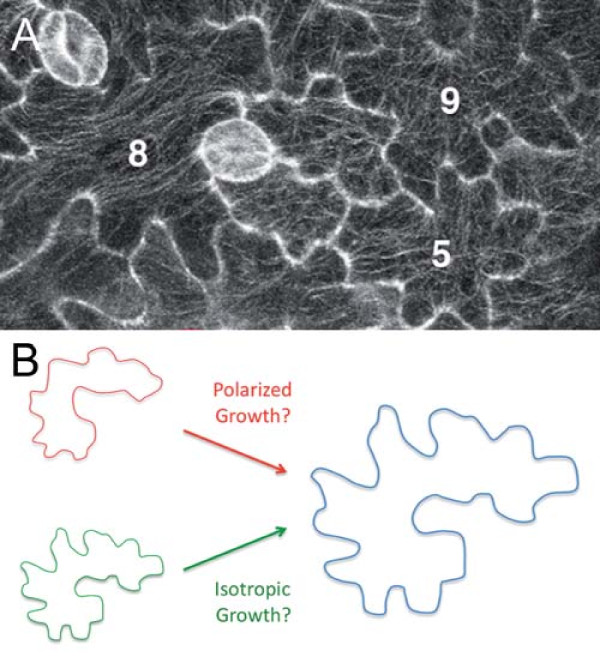
**A jigsaw puzzle in the leaf epidermis**. **(A) **GFP:TUB6 labeled cortical microtubule arrays in cotyledon pavement cells 3 days after germination. Individual cells are numbered. Reproduced from Zhang *et al. *[2]. **(B) **Continuous polarized growth or isotropic growth from a lobed initial cell can lead to the typical pavement cell shape.

Leaves (including cotyledons, the embryonic 'seed leaves' used in this study) exhibit a stereotypical feature in their epidermis: most of the epidermal cells, called 'pavement cells', exhibit 'lobes' and 'necks' that interlock with those of adjacent cells, so they are tightly stuck to each other, as observed in a jigsaw puzzle (Figure 2a). Several theories have been proposed as to why such a histological structure would emerge during evolution, including the need to build a mechanically strong epidermis. Equally interesting is the question of how such a structure can be generated and maintained during growth. In particular, as a lobe always emerges opposite a neck, how can each cell control its growth tightly enough not to generate gaps between growing cells?

In plants, diffuse growth is the term used to describe the growth of individual cells within a tissue: each contiguous cell is glued to its neighbor by the middle lamella of the cell walls, and thus growth of a given cell is subject to mechanical stress generated by the growth of its neighbors. A few other cell types (typically pollen tubes and root hairs) undergo a different growth mode, called tip-growth, in which growth occurs with no physical connection to neighboring cells, and through the elongation of the cell tip. While the pavement cells obviously have all the features associated with the diffuse growth mode, it has been proposed that the lobe domains of these cells behave like tip-growing regions, notably by expanding at a greater rate than the rest of the cell (Figure 2b). Molecular evidence supports this model: as in tip-growing cells, actin accumulates at the tip of a lobe, consistent with a local polarized elongation. Cortical microtubules, as viewed from the top, form a ring at the base of lobes, and, via their impact on cellulose deposition, would thus restrict growth locally and generate necks. Lastly, the organization of the cytoskeleton in both pavement cell lobes and tip-growing cells involves the polar localization of Rho of Plants (ROP) proteins, and the ARP2/3 and SCAR/WAVE complexes [2,3].

## A quantitative analysis of shape change during growth

To check the dynamics of this system, Zhang *et al. *[2] performed a careful quantitative analysis of cotyledon pavement cell shape over time, and showed that lobes are initiated early on, within the first 3 days after germination. Most of pavement cell growth occurs after this time and is in fact close to isotropic (uniform in all directions); to reach this conclusion, the authors measured both the top wall area and the length of each cell side, and the measurements were subjected to a simple linear regression analysis. In other words, the puzzle shape of the pavement cells becomes more obvious after day 3 not because of continuous polarized growth, but because of a homothetic magnification of a shape already existing at day 3 (Figure 2b).

Interestingly, and consistent with these data, there was not always a clear correlation between the pattern of cortical microtubules, as viewed from the top, and cell shape after day 3 (Figure 2a). Venturing beyond this classical viewpoint of the microtubules, Zhang and collaborators used fixed tissues (to mildly separate each cell and access the cell sides) as well as single cell transformation (through bombardment) to image the microtubules on the anticlinal wall of the cells. From these data, microtubules were shown to populate the lobe domain, in contrast to the predominant model. This questions the supposed specific role of the microtubules in limiting growth in the necks and, conversely, it also suggests a contribution of the microtubules in driving lobe growth, in addition to actin.

One conclusion from this study is that growth of the young leaf epidermis is truly diffuse after day 3, and this can be correlated with reducing the risk of generating shearing stress between neighboring cells. While this study also leaves a lot of questions to address, it pinpoints the need for quantitative approaches in order to generate reliable hypotheses and drive experimental design. In this case, two very distinct processes, involving different growth modes and cytoskeleton dynamics, seem to control cell morphogenesis in the epidermis.

The quantitative analysis also revealed new unexpected behaviors: in contrast to growth in the XY plane, growth in height, a variable that had not been quantified and correlated to pavement cell shape before, followed a non-linear behavior after day 3. Although it is unclear how this could be achieved, these data suggest that growth in the Z direction might have a more important role in pavement cell morphogenesis than previously anticipated.

## Frontiers of quantitative imaging

More generally, the development of quantitative approaches in four dimensions is becoming crucial. Several recent technical breakthroughs, such as light sheet-based technologies, have enabled the imaging of cells in developing organisms in three dimensions over time at very high resolution. Keller and collaborators [4], for instance, managed to correlate patterns of cell migration and division with morphogenesis, prior to the formation of the dorso-ventral axis in zebrafish embryos (see also [5]). In plants, imaging epoxyresin replicas of tissues with scanning electron microscopy at different angles allowed the precise segmentation of cell shapes in the epidermis over time, notably revealing the existence of regions with characteristic growth and curvature features in the shoot apical meristem [6]. More recently, Fernandez *et al. *[7] developed a three angle confocal-based pipeline called MARS-ALT that monitors the elongation of each cell wall in a three-dimensional plant object such as a floral bud.

The increasing resolution of the description enabled by these and forthcoming techniques is likely to set new standards in quantitative imaging. In this respect, it is notable that several seminal papers are purely descriptive in nature. For instance, using a modified TIRF (total internal reflection fluorescence) protocol (variable-angle epifluorescence illumination), Staiger and collaborators [8] were able to describe single actin filament dynamics at the cell cortex in living hypocotyl cells, revealing striking plant-specific filament behaviors. While descriptive approaches can be viewed as either too laborious or not goal oriented enough, the emergence of novel testable concepts using quantitative strategies strongly suggests otherwise.
